# Green Synthesis of Silver Nanoparticles Using the Cell-Free Supernatant of *Haematococcus pluvialis* Culture

**DOI:** 10.3390/ma17010187

**Published:** 2023-12-29

**Authors:** Maria G. Savvidou, Evgenia Kontari, Styliani Kalantzi, Diomi Mamma

**Affiliations:** 1 Biotechnology Laboratory, School of Chemical Engineering, National Technical University of Athens, Zografou Campus, 9 Iroon Polytechniou Str, 15780 Athens, Greece or maria_theognosia.savvidou@tufts.edu (M.G.S.); evgeniakontari@gmail.com (E.K.); stykalan@chemeng.ntua.gr (S.K.); 2 Department of Biomedical Engineering, Tufts University, Medford, MA 02155, USA

**Keywords:** green synthesis, microalgae *Haematococcus pluvialis*, cell-free supernatant, silver nanoparticles, synthesis conditions, antibacterial activity

## Abstract

The green synthesis of silver nanoparticles (AgNPs) using the cell-free supernatant of a *Haematococcus pluvialis* culture (CFS) was implemented in the current study, under illumination conditions. The reduction of Ag^+^ to AgNPs by the CFS could be described by a pseudo-first-order kinetic equation at the temperature range tested. A high reaction rate during synthesis and stable AgNPs were obtained at 45 °C, while an alkaline pH (pH = 11.0) and a AgNO_3_ aqueous solution to CFS ratio of 90:10 (*v*/*v*) proved to be the most effective conditions in AgNPs synthesis. A metal precursor (AgNO_3_) at the concentration range tested (1–5 mM) was the limited reactant in the synthesis process. The synthesis of AgNPs was accomplished under static and agitated conditions. Continuous stirring enhanced the rate of reaction but induced aggregation at prolonged incubation times. Zeta potential and polydispersity index measurements indicated stable AgNPs and the majority of AgNPs formation occurred in the monodisperse phase. The X-ray diffraction (XRD) pattern revealed the face-centered cubic structure of the formed AgNPs, while TEM analysis revealed that the AgNPs were of a quasi-spherical shape with a size from 30 to 50 nm. The long-term stability of the AgNPs could be achieved in darkness and at 4 °C. In addition, the synthesized nanoparticles showed antibacterial activity against *Escherichia coli*.

## 1. Introduction

Nanotechnology is an emerging field of research, with numerous applications in science and technology, especially in the development of different nanomaterials and nanoparticles. Nanoparticles (NPs) are small particles with a size from 1 nm to 100 nm. Their size is approximately 100 to 10,000 times smaller than that of biological molecules such as enzymes, receptors, and antibodies [1]. Owing to their ultra-small size, large surface area, and high surface activity, NPs exhibit fascinating size-/structure-dependent properties and superiorities (e.g., electrical, magnetic, optical, chemical, biological) compared to their corresponding bulk materials [2,3]. 

Top-down and bottom-up methods are two means to achieve nanoscale components. In the top-down approach, the synthesis of nanoparticles takes place by breaking down the bulk material into fine-sized particles. The top-down strategy mostly refers to the physical methods of NPs synthesis, using techniques such as grinding, milling, sputtering, laser ablation, evaporation–condensation, and the hydrothermal method. However, the low production rate, expensive operations, and high energy consumption are the major limitations of these processes. The bottom-up approach involves the formation of nanostructures from smaller atoms and molecules. Chemical reduction is mostly used in this approach, sometimes combined with a capping agent for the stabilization of the synthesized NPs. The main disadvantages of the chemical methods for the production of NPs are the high cost and the production of toxic byproducts to both human health and the environment [2,4,5,6,7,8].

The green synthesis of NPs is a bottom-up approach that utilizes a biological system or its parts as a reductant for the synthesis of NPs. Green synthesis has many advantages compared to chemical methods: it is simple, easy to scale up, non-toxic, pollution-free, environmentally friendly, economical, and more sustainable [2,9].

Among the different NPs, silver nanoparticles (AgNPs) have emerged with leading contributions in various fields, including healthcare, cosmetics, agriculture, water treatment, biosensors, textiles, and the food industry [10,11]. Their specificity and significance arise from their antibacterial, antioxidant, anti-inflammatory, antiviral, and even cytotoxic and catalytic properties [12,13,14]. The green synthesis of AgNPs mediated by different biological entities has gained increased interest. Various organisms, such as fungi, algae, bacteria, and plants, as well as their metabolites, have been used in AgNPs synthesis with diverse sizes and shapes, exhibiting, in general, the same properties as AgNPs synthesized by physicochemical methods [13,15,16,17,18]. Microalgae are a diverse group of unicellular photosynthetic organisms, including eukaryotic protists, prokaryotic cyanobacteria, and blue-green algae. They have the ability to grow rapidly in different aquatic habitats and adapt to adverse environmental conditions. Microalgae have been used in food, cosmetics, biofuels, and the nutraceutical and pharmaceutical industries, as well as in wastewaters’ bioremediation and CO_2_ removal [19]. A wide range of biomolecules, such as pigments, lipids, polysaccharides, biopolymers, proteins, and vitamins, can be found in microalgae [20,21]. These biomolecules are responsible for the reduction of the silver ions and also can serve as capping agents, whose role is to prevent agglomeration, reduce toxicity, and increase the stability of nanoparticles [17,22]. The bioreduction of silver ions to their corresponding nanoparticles using microalgae can be achieved either intracellularly or extracellularly. Intracellular synthesis takes place inside the cell. It is well known that microalgae have developed effective defensive mechanisms to counter the damaging effects of heavy metal ions that have accumulated in cellular organelles. Reducing agents (NADPH, NADPH-dependent reductase, nitrogenase) produced through different metabolic pathways, such as photosynthesis, respiration, and nitrogen fixation, can carry out the biosynthesis of metal nanoparticles [15,22,23,24]. Extracellular synthesis takes place outside the cell and, in this procedure, cell extracts (aqueous or organic), cell-free supernatants, or specific biomolecules obtained from the culture of microalgae, as well as whole cells, can be used. In this case, pigments, polysaccharides, peptides, and enzymes present in the cell extract or cell-free supernatant reduce silver ions to nanoparticles [17,22]. An advantage of extracellular synthesis compared to intracellular synthesis is that no cell lysis is required to obtain the nanoparticles [18].

The size, shape, and stability of the microalgae-mediated extracellularly synthesized AgNPs is affected significantly by the synthesis conditions, such as illumination, the pH of the reaction medium, the temperature, the metal precursor concentration, the incubation time/reaction time, the ratio of reagents, and the stirring conditions. Furthermore, apart from the synthesis conditions, the shape of the biosynthesized AgNPs was reported to be dependent on the microalga strain used [17,25]. 

*Haematococcus pluvialis* is a freshwater unicellular green microalga belonging to the class Chlorophyceae. *H. pluvialis* accumulates large quantities of astaxanthin, a natural antioxidant, compared to other organisms [26,27]. Taking into consideration the importance of this natural product, current research is directed towards enhancing astaxanthin production by manipulating the bioprocess conditions (nitrogen deficiency, high light illumination, elevated carbon dioxide levels, salinity stress) or using small-molecule chemicals to stimulate astaxanthin accumulation [28]. Recently, it was reported that the use of AgNPs improved astaxanthin production by *H. pluvialis* [29,30].

**Figure 1:**
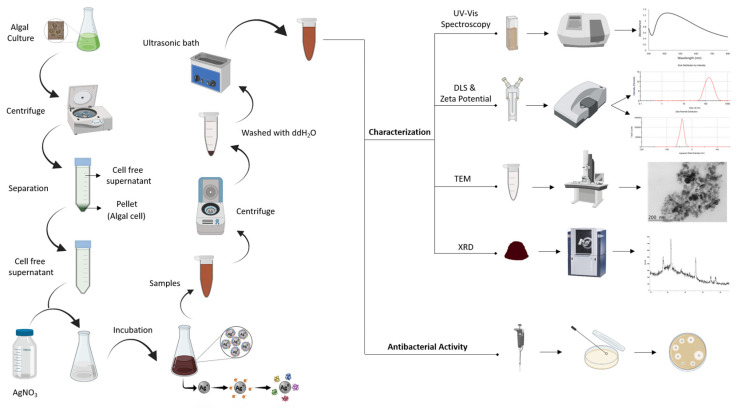
Schematic representation of the AgNPs synthesis workflow (created with BioRender.com).

The present study was undertaken to investigate, for the first time, the extracellular synthesis of AgNPs mediated by the cell-free supernatant (CFS) of a *H. pluvialis* culture. The effect of the synthesis conditions, such as illumination, the pH of the reaction medium, the temperature, the metal precursor concentration, the metal precursor to cell-free supernatant ratio, the stirring conditions, and the incubation time/reaction time, were examined. The synthesized nanoparticles were characterized via UV–vis spectroscopy, dynamic light scattering (DLS), X-ray diffraction (XRD), and transmission electron microscopy (TEM). Furthermore, the antibacterial activity of the biosynthesized AgNPs was examined (Figure 1). 

## 2. Materials and Methods

### 2.1. Organism and Growth Conditions

The *Haematococcus pluvialis* strain (CCAP 34/6) was purchased from the Culture Collection of Algae and Protozoa (Dunbeg, UK). The strain was grown as described previously [31]. NaHCO_3_ was added at a concentration of 1 g/L and the pH was adjusted to 7.5. The cultures were incubated in an orbital shaker (Zhicheng Analytical Instruments Manufacturing Co., Ltd., ZHWY-211C Incubator Shaker, Shanghai, China) at 100 rpm and 23 °C, under illumination with white LED lamps at 80 μmol photon m^−2^ s^−1^ with a 24-h light cycle. The growth of *H. pluvialis* was monitored by measuring the OD at 680 nm using a spectrophotometer (S-20 Spectrophotometer, Boeco, Hamburg, Germany). 

Cultures at the exponential phase were centrifuged at 4500 rpm for 10 min at 4 °C (Andreas Hettich GmbH & Co. KG, Rotanta 460 R, Tuttlingen, Germany) to obtain the cell-free supernatant (CFS), which was used as the bioreducing agent in AgNPs production.

### 2.2. Synthesis of AgNPs

The AgNPs were synthesized using AgNO_3_ as the metal precursor. Initially, the effect of the illumination conditions on AgNPs formation was investigated. AgNPs synthesis in the dark was conducted in Erlenmeyer flasks wrapped with double aluminum foil. Continuous illumination during synthesis was provided by white LED lamps at 80 μmol photon m^−2^ s^−1^. The synthesis conditions were the following: total volume of the reaction medium 50 mL, 1 mM aqueous solution of AgNO_3_ adjusted to pH = 8.0, AgNO_3_ aqueous solution to CFS ratio 90:10 (*v*/*v*), temperature 25 °C, stirring at 180 rpm for 15 min, followed by stirring at 80 rpm. 

Different parameters affecting the AgNPs’ synthesis were consecutively studied, namely the pH (5.0, 7.0, 8.0, 9.0 and 11.0), temperature (25, 35, 45 and 55 °C), concentration of the metal precursor (1, 2, 3, 4 and 5 mM), AgNO_3_ aqueous solution to CFS ratio (95:5, 90:10 and 85:15, *v*/*v*) and agitation (static conditions, continuous stirring at 180 rpm, stirring at 180 rpm for 15 min followed by static conditions, and stirring at 180 rpm for 15 min followed by stirring at 80 rpm). In the case of the pH study, the corresponding pH of the AgNO_3_ solutions was adjusted to pH 5.0, 7.0, 8.0, 9.0, and 11.0 using 1 N HCl or 1 N NaOH [32].

The one factor at a time method (OFAT) was applied when studying the effect of the pH, T, concentration of the metal precursor, AgNO_3_ aqueous solution to CFS ratio, and agitation. In this method, the impact of a change in one factor is studied when all the other factors are kept constant.

Samples were withdrawn aseptically, at different time intervals (15 min, 1, 3, 6, 24 h), for further analysis. Each experimental condition was examined in duplicate.

### 2.3. Characterization of AgNPs

The reduction of Ag^+^ ions was monitored by UV–vis spectrophotometry (Hitachi, U-5100 Spectrophotometer, Tokyo, Japan) in the wavelength range of 300–800 nm, at different time intervals. 

The surface charge of the AgNPs was determined by measuring the zeta potential, using the Zetasizer Nano ZS (Malvern Zetasizer Nano ZS, Malvern, UK) and the software provided by the manufacturer. Prior to analysis, samples (2 mL) were centrifuged (4500 rpm for 10 min at 4 °C) (Hettich, Rotanta 460 R), the supernatant was discarded, and the pellet (AgNPs) washed two times with double-distilled water (ddH_2_O) and finally re-suspended in ddH_2_O (final volume 2 mL). The solution was sonicated in an ultrasonic bath (EMAG Technologies, Emmi-30HC, Ann Arbor, MI, USA) to disaggregate the agglomerated particles. 

Powder X-ray diffraction patterns were recorded using a D8 Advance Diffractometer (Bruker, Billerica, MA, USA) with a CuKa radiation source (λ = 1.5406 Å). The patterns were recorded from 20 to 80° 2θ at a constant rate of 0.05° s^−1^. 

TEM analysis was performed to determine the size and the shape of the synthesized AgNPs. Analysis was performed in a high-resolution JEOL JEM-2100 LaB_6_ (JEOL, Road Peabody, MA, USA) transmission electron microscope (HRTEM), operating at an accelerating voltage of 200 kV. Prior to analysis, samples (~0.2 mL) were suspended in ddH_2_O and treated with ultrasound to disaggregate the agglomerated particles.

### 2.4. Long-Term Stability of the Biosynthesized AgNPs

The stability of biosynthesized AgNPs was studied in the dark at two different storage temperatures, ambient and 4 °C. Following synthesis, the reaction mixture was centrifuged (4500 rpm for 10 min at 4 °C) (Hettich, Rotanta 460 R), the supernatant was discarded, and the pellet (AgNPs) was washed two times with double-distilled water (ddH_2_O) and finally re-suspended in ddH_2_O. The aqueous solutions were stored in the dark at the abovementioned temperatures for up two months. Samples were withdrawn at different time intervals. The stability was monitored by UV–vis spectrophotometry and zeta potential measurements.

### 2.5. Antibacterial Activity of AgNPs

The antibacterial activity of the biosynthesized AgNPs was evaluated against the Gram-negative bacterium *E. coli* by the disk diffusion method. *E. coli* was grown on Luria–Bertani (LB) liquid medium, in an orbital shaker, at 37 °C for 24 h. LB agar plates were inoculated with 0.1 mL of bacterial culture at a cell density of 10^6^ cells/mL. Discs (5 mm in diameter), prepared using Whatman No. 1 filter paper, were impregnated with 50 μL of the AgNPs aqueous solution. Following air drying, the discs were placed over the inoculated agar surface. The inoculated plates were incubated at 37 °C for 24 h, after which the plates were checked for the existence of inhibition zones. The cell-free supernatant of the *H. pluvialis* culture as well as 1 mM aqueous solution of AgNO_3_ were also tested against *E. coli*.

## 3. Results and Discussion

### 3.1. Biosynthesis of AgNPs by the Cell-Free Supernatant of H. pluvialis Culture 

The algae-mediated biosynthesis of AgNPs can be conducted intra- or extracellularly, by taking advantage of the wide range of biomolecules found inside the microalgae cell or of those excreted in the culture medium. Several investigators have applied the extracellular synthesis of AgNPs using microalgae cell extracts, extracellular polymeric substances (EPS), or the CFS of microalgae cultures [33,34,35,36,37,38,39,40,41,42,43,44].

In the present study, the CFS of the *H. pluvialis* culture was used as a source of bioreducing agents. The formation of AgNPs by the CFS of the *H. pluvialis* culture was investigated under various conditions affecting the synthesis, such as the illumination conditions, pH, temperature, concentration of the metal precursor, AgNO_3_ aqueous solution to CFS ratio, and stirring. A time-dependent analysis was carried out for all of the above parameters. In general, an increase in reaction (incubation) time could increase the number of nanoparticles; however, upon extending the reaction time, the agglomeration of AgNPs occurred.

**Figure 2:**
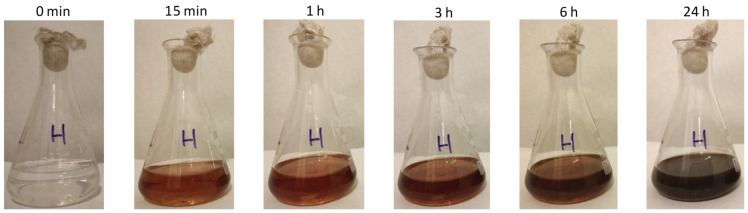
Color change of the reaction medium at different time points.

In all cases, the addition of the AgNO_3_ solution into the CFS of the *H. pluvialis* culture caused a color change from colorless to red brown and finally to dark brown, which was indicative of particle formation (Figure 2) [36,37]. Furthermore, the synthesis of AgNPs was confirmed by UV–vis absorption spectroscopy.

In general, the UV–vis spectra of the AgNPs have a strong band in the visible region ranging from 390 to 470 nm, corresponding to the surface plasmon resonance (SPR) of the AgNPs. AgNPs have a small size and spherical shape when the SPR band is located between 390–420 nm and 410–450 nm [45,46]. The specific position of the peaks depends on the experimental conditions applied—for instance, the type of bioreducing agent, concentration, temperature, pH, etc.—but the SPR band peaks of the AgNPs always fall within the abovementioned range [47]. In addition, the CFS and AgNO_3_ solution did not show any SPR absorption bands, verifying that the only SPR bands observed were the result of the reduction of Ag^+^ to AgNPs.

#### 3.1.1. Effect of Illumination Conditions

**Figure 3:**
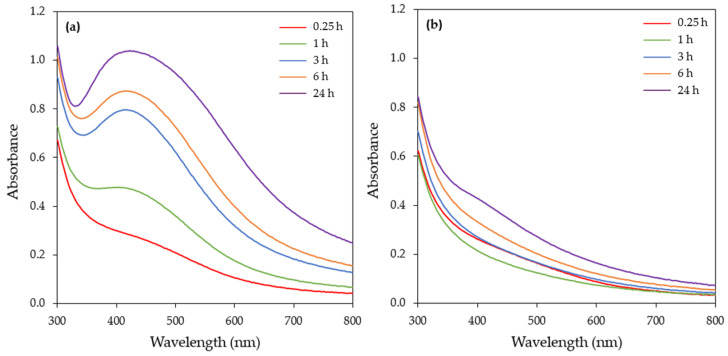
UV–visible absorption spectra of the AgNPs biosynthesized by the CFS of *H. pluvialis* culture under (**a**) illumination and (**b**) dark conditions (reaction time 24 h). (Conditions: pH = 8.0, T = 25 °C, AgNO_3_ concentration: 1 mM, AgNO_3_ aqueous solution to CFS ratio: 90:10 (*v*/*v*), stirring: 180 rpm for 15 min followed by stirring at 80 rpm).

Illumination is a critical physical factor that can affect the synthesis of AgNPs and thus the synthesis of AgNPs from the CFS of the *H. pluvialis* culture was initially examined under dark and illuminated conditions. The UV–vis spectra of the samples (Figure 3) were recorded at various time intervals (15 min, 1, 3, 6, 24 h). The UV–vis spectra of the illuminated samples show a strong single peak around 420 nm, which is attributed to the SPR band of AgNPs. 

A continuous increase in the SPR band intensity with respect to time was observed, reaching its maximum after 24 h of reaction. Under dark conditions, no synthesis of AgNPs was observed as the UV–vis spectra did not exhibit any SPR absorption band at up to 6 h of reaction time. A barely visible SPR band was recorded in 24 h (Figure 3b). 

As a result, the illumination condition was chosen for further experimentation. Several studies have demonstrated the importance of light during AgNPs synthesis mediated by extracts of microalgae cells or CFS. Patel et al. [35] tested the CFS of eight microalgae species for the synthesis of AgNPs both under light and dark reaction conditions and reported that, in most of the cases, AgNPs were formed in light but not in dark conditions. Husain et al. [41] also reported that the synthesis of AgNPs from the cell extracts of 30 different cyanobacterial species, including *Microchaete* sp. NCCU-342, *Phormidium* sp. NCCU-104, *Cylindrospermum stagnale* NCCU, and *Hapalosiphon fontinalis* NCCU-339, was conducted under illumination. Furthermore, the synthesis of AgNPs using the EPS of *Chlamydomonas reinhardtii* or the aqueous cell extract of *Neochloris oleoabundans* or *Dunaliella salina* was feasible only in the presence of light [39,42,43,44] (Table 1).

#### 3.1.2. Effect of Temperature

**Figure 4:**
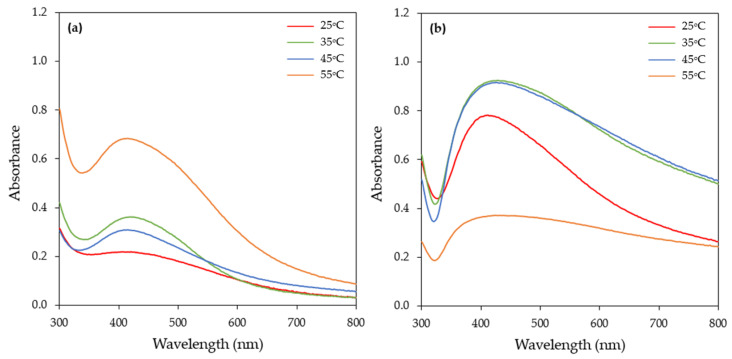
UV–visible absorption spectra of AgNPs biosynthesized by the CFS of *H. pluvialis* culture at different temperatures and for reaction times of (**a**) 1 h and (**b**) 24 h. (Conditions: illumination, pH = 8.0, AgNO_3_ concentration: 1 mM, AgNO_3_ aqueous solution to CFS ratio: 90:10 (*v*/*v*), stirring: 180 rpm for 15 min followed by stirring at 80 rpm).

Temperature is an important parameter in AgNPs synthesis, and its alteration can affect the shape and size of AgNPs [17,24]. The synthesis of AgNPs by the CFS of the *H. pluvialis* culture was investigated at four different temperatures: 25, 35, 45, and 55 °C. The UV–vis spectra of the synthesized AgNPs showed a single peak at around 420 nm at all temperatures tested (Figure 4). The rapid production of AgNPs, within 60 min, was observed at 55 °C (Figure 4a), while, for reaction times greater than 3 h, the SPR band broadened towards longer wavelengths (Figure 4b and Figure S1), which was an indication of the aggregation of the particles. 

**Figure 5:**
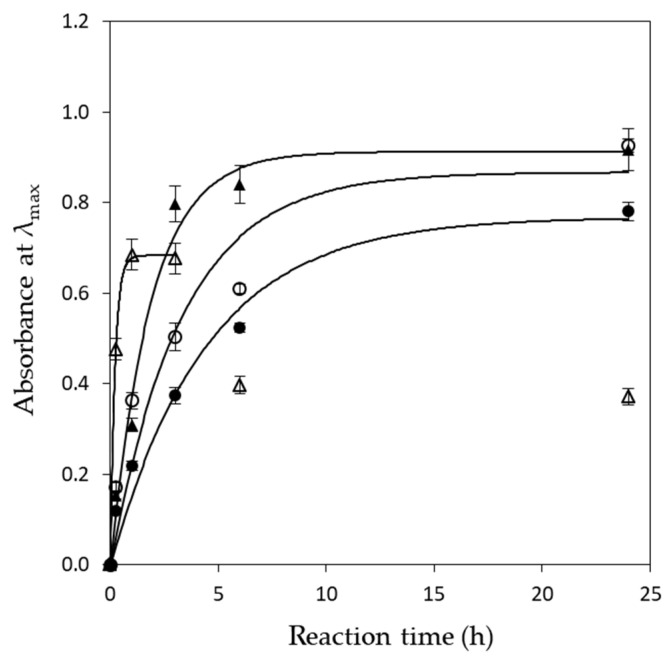
Maximum UV–vis absorbance of the produced AgNPs vs. time. Symbols (●) 25 °C, (○) 35 °C, (▲) 45 °C and (△) 55 °C. Solid line represents model fitting. At 55 °C, the model was fitted up to 3 h.

On the other hand, at the lower temperature tested (25 °C), the intensity of the SPR band at 60 min was very low (Figure 4a), indicating that silver ion reduction rate under these conditions was slower, while, upon extending the reaction time, the absorbance of AgNPs continuously increased (Figure 5). The same pattern was observed when the reaction was conducted at 35 °C. At 45 °C, the absorbance quickly increased, subsequently slowed, and finally reached a balance (Figure 5).

The reduction of Ag^+^ to AgNPs by the CFS of the *H. pluvialis* culture can be described by a pseudo-first-order kinetic equation (Equation (1)) [50]:

(1) $$ln\left(\frac{{\left[{Ag}^{+}\right]}_{t}}{{\left[{Ag}^{+}\right]}_{0}}\right)=-kt$$

where [*Ag^+^*]_0_ and [*Ag^+^*]*_t_* are the concentrations of *Ag^+^* at time *0* and *t*, respectively; *k* is the pseudo-first-order rate constant.

According to the principle of mass conservation [51],

(2) $$n\cdot {\left[{Ag}_{n}\right]}_{t}+{\left[{Ag}^{+}\right]}_{t}={\left[{Ag}^{+}\right]}_{0}$$

where *n* is the average agglomeration number of AgNPs and [*Ag_n_*]*_t_* is the concentration of AgNPs at time *t*.

Solving Equation (2) for [*Ag^+^*]*_t_* and substituting it into Equation (1) results in

(3) $$-ln\left(1-\frac{{n\left[{Ag}_{n}\right]}_{t}}{{\left[{Ag}^{+}\right]}_{0}}\right)=kt$$

The absorbance of AgNPs follows Lambert–Beer’s law [51]:

(4) $$Abs=a\cdot {\left[{Ag}_{n}\right]}_{t}$$

where *Abs* is the maximum absorbance value of the AgNPs and *a* is a constant that includes both the extinction coefficient and optical path length.

Substituting this into Equation (3) results in

(5) $$Abs=m\cdot {\left[{Ag}^{+}\right]}_{0}\left[1-e^{-kt}\right]$$

where *m* is a constant ($m=\frac{a}{n}$).

Equation (5) describes the change in absorbance of the produced AgNPs with time. Fitting the data presented in Figure 5 with Equation (5), the values of the pseudo-first-order rate constant (*k*) could be calculated. In all cases, the model (Equation (5)) fitted very well with the experimental data (0.96 < R^2^ < 0.99). The *k* values were found as 0.224, 0.301, 0.535, and 4.803 h^−1^ at 25, 35, 45, and 55 °C, respectively. The results were in line with the observations made from the UV–vis spectra and indicated that AgNPs synthesis was faster at 55 °C (higher *k* value). 

A wide range of temperatures, from room temperature up to 95 °C, have been reported in the literature for the optimal synthesis of AgNPs by microalgae. The temperature employed in the different studies is dependent on the microalgae strain as well as on the method used for the synthesis (intra- or extracellularly) (Table 1). Concerning extracellular synthesis, Prasad et al. [48] used an extract of the marine algae *Cystophora moniliformis* for the synthesis of AgNPs and reported that, at temperatures lower than 65 °C, the AgNPs produced were spherical, with a size range of 50–100 nm, while, at higher temperatures of up to 95 °C, the size of the AgNPs produced was greater than 2 μm. Aboelfetoh et al. [52] conducted the synthesis of AgNPs with an extract of the green algae *Caulerpa serrulata* at a temperature range of 27–95 °C and reported enhanced reaction rates at 95 °C and the formation of smaller nanoparticles. The reduction in the AgNPs’ size when produced at higher temperatures could be attributed to the fact that when the reaction temperature increases, the reaction rate increases and, as a result, the formation of nuclei is favored over the secondary reduction process on the surfaces of pre-formed nuclei [25].

A high temperature (90 °C) has also been applied in AgNPs synthesis with the EPS of *Chlorella pyrenoidosa* [36]. The synthesis of AgNPs by the cell-free aqueous extract of *Microchaete* NCCU-342 as well as by the CFS of a *Chlorella vulgaris* culture was conducted at 50 °C [25,53], and AgNPs production by the cell-free extract of the latter microalgae was optimal at room temperature [34]. 

#### 3.1.3. Effect of pH

A change in the pH can influence the size and shape of biogenic nanoparticles. The pH alters the electrical charge of the biomolecules, regulating their ability to reduce metal ions [17,49]. Five different pH values (5, 7, 8, 9, and 11) were tested, with pH = 11.0 being the most effective in AgNPs synthesis, even from 15 min of incubation, as indicated from the intensity of the SPR band (Figure S2). 

**Figure 6:**
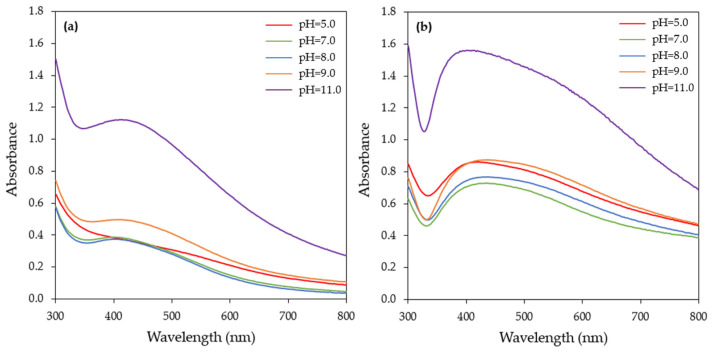
UV–visible absorption spectra of AgNPs biosynthesized by the CFS of *H. pluvialis* culture at different pHs and for reaction times of (**a**) 1 h and (**b**) 24 h. (Conditions: illumination, T = 45 °C, AgNO_3_ concentration: 1 mM, AgNO_3_ aqueous solution to CFS ratio: 90:10 (*v*/*v*), stirring: 180 rpm for 15 min followed by stirring at 80 rpm).

The alkaline pH used in the present study seemed to accelerate the reduction rate of Ag^+^ to AgNPs. This could be probably attributed to the increase in electrostatic interactions between the negatively charged functional groups present in the CFS and the positively charged silver ions. AgNPs synthesis started also at 15 min, when the pH of the reaction medium was 9.0, but at a lower rate compared to pH = 11.0. On the other hand, at pH values of 5.0, 7.0, and 8.0, the SPR band was barely visible at 15 min (Figure S2) and started to appear after 1 h of reaction (Figure 6a). In all pH levels tested, a continuous increase in absorbance over time was observed, reaching its maximum after 24 h (Figure 6b). It also should be noted that when extending the reaction time, the SPR band broadened towards a longer wavelength (Figure 6b), which indicated the aggregation of the nanoparticles.

Several authors have reported that microalgae extracts exhibit better reducing power under alkaline pHs (Table 1). For instance, Mora-Godínez et al. [32] reported that the optimum pH for AgNPs synthesis using the CFS of a *Desmodesmus abundans* RSM culture was 11.0, while, at a lower pH (5.0 and 7.5), no SPR absorption band was observed. Similarly, AgNPs synthesis by the cell-free extract of *C. vulgaris*, the extract of *C. serrulata*, and the EPS of *Shewanella oneidensis* MR-1 was achieved at alkaline pHs, as indicated by a strong SPR band [34,50,52]. On the other hand, Hamouda et al. [49] showed that the optimum pH for AgNPs synthesis by the aqueous extract of the *Oscillatoria limnetica* fresh biomass was 6.7, and when the pH was altered to 4.7 or 5.7, AgNPs synthesis was completely inhibited. Furthermore, Bao et al. [43] reported that AgNPs production by the cell extract of green algae *N. oleoabundans* was maximized at pH 5.0. 

#### 3.1.4. Effect of Metal Precursor Concentration

**Figure 7:**
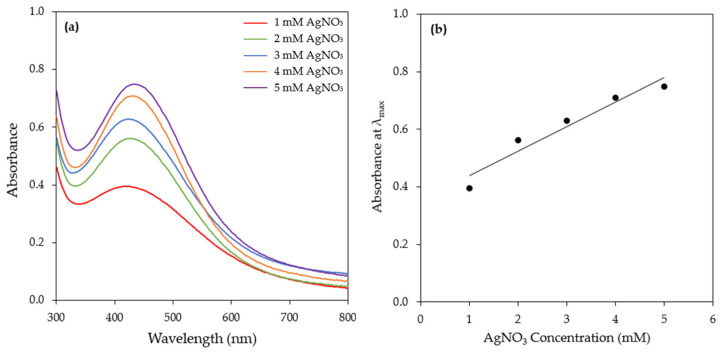
(**a**) UV–visible absorption spectra of AgNPs biosynthesized by the CFS of *H. pluvialis* culture at different AgNO_3_ concentrations (reaction time 15 min). (Conditions: illumination, T = 45 °C, pH = 11.0, AgNO_3_ aqueous solution to CFS ratio: 90:10 (*v*/*v*), stirring: 180 rpm for 15 min followed by stirring at 80 rpm). (**b**) Linear relationship between AgNO_3_ concentration and absorbance at λ_max_ (420 nm), $ABS\;(at\;\lambda_{max})=0.0854*C_{{AgNO}_{3}}+0.3522$, (R^2^ = 0.97, *p* = 0.004).

The effect of the precursor (AgNO_3_) concentration (1–5 mM) on AgNPs synthesis was investigated (Figure 7a and Figure S3). An increase in the AgNO_3_ concentration resulted in an increase in the intensity of the SPR band of AgNPs. More specifically, even from 15 min of the reaction, the SPR band intensity increased linearly with increasing concentrations of AgNO_3_, indicating that AgNO_3_ was the limiting reactant in the reduction process (Figure 7b). This observation implies that the content of bioreducing agents in the CFS of the *H. pluvialis* culture was high and that by supplying more Ag^+^ to the reaction medium, more AgNPs could be produced. Linearity was observed at all time points tested (Figure S4). 

Similarly to the present study, Rahman et al. [54] used three different precursor concentrations (0.125, 0.650, and 1.250 mM) for the synthesis of AgNPs by *C. reinhardtii* and they reported that the SPR band intensity depended linearly on the initial AgNO_3_ concentration. Furthermore, Hamouda et al. [49] observed that during the synthesis of AgNPs by *O. limnetica*, the increase in the SPR band intensity followed a silver nitrate dose-responding manner. The synthesis of AgNPs by the cell extract of *N. oleoabundans* increased significantly when the AgNO_3_ concentration increased from 0.2 to 0.4 mM; however, a decline was observed with a further increase in the AgNO_3_ concentration [43]. Similarly, Husain et al. [25] reported that at a AgNO_3_ concentration higher than 1 mM, a decrease in SPR band intensity was observed. Rajkumar et al. [34] reported that AgNPs synthesis by the cell-free extract of *C. vulgaris* was achieved at a metal precursor concentration of 3 mM, while, at lower or higher concentrations, no prominent SPR band was observed.

#### 3.1.5. Effect of AgNO_3_ Aqueous Solution tο Cell-Free Supernatant Ratio

**Figure 8:**
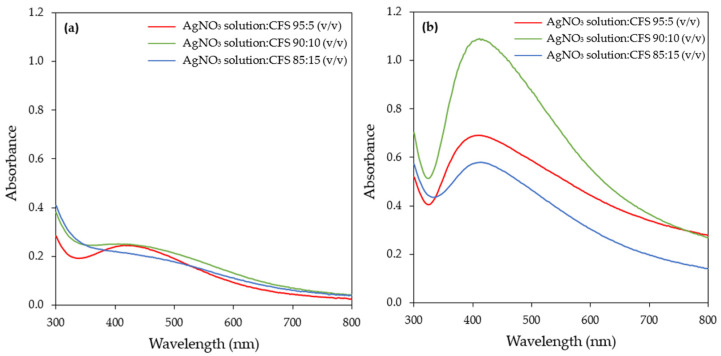
UV–visible absorption spectra of AgNPs biosynthesized by the CFS of *H. pluvialis* culture at different AgNO_3_ aqueous solution to CFS ratios and for reaction times of (**a**) 1 h and (**b**) 24 h. (Conditions: illumination, T = 45 °C, pH = 11.0, AgNO_3_ concentration: 1 mM, stirring: 180 rpm for 15 min followed by stirring at 80 rpm).

The AgNO_3_ aqueous solution tο bioreducing agent ratio is an important parameter that influences both the yield and morphological characteristics of the synthesized AgNPs. Three AgNO_3_ aqueous solution tο CFS ratios were studied, namely 95:5, 90:10, and 85:15 (*v*/*v*). The UV–vis spectra of the samples recorded at 1 and 24 h of reaction are depicted in Figure 8. The SPR band intensity increased with time at all ratios tested and exhibited its maximum at 24 h (Figure 8 and Figure S5). At this time point, the ratio of 90:10 *v*/*v* exhibited the highest intensity of the SPR band, while a reduction in the intensity of the SPR band was observed with a further increase in the AgNO_3_ aqueous solution tο CFS ratio (85:15, *v*/*v*). 

Similar observations were made by several investigators when they studied the effect of the AgNO_3_ aqueous solution tο bioreducing agent ratio. It seems that there is an optimal value of this factor above which a reduction in the intensity of the SPR band occurs. This has been attributed to the aggregation of the nanoparticles at a higher bioreducing agent concentration [52,55,56].

#### 3.1.6. Effect of Stirring

In order to evaluate the effect of stirring on the rate of AgNPs formation, synthesis was carried out under four different agitation conditions: static conditions, continuous stirring at 180 rpm, stirring at 180 rpm for 15 min followed by static conditions, and stirring at 180 rpm for 15 min followed by stirring at 80 rpm. In general, stirring provides a more homogenous suspension environment, hence facilitating the mass transfer process and thus the AgNPs’ growth. 

**Figure 9:**
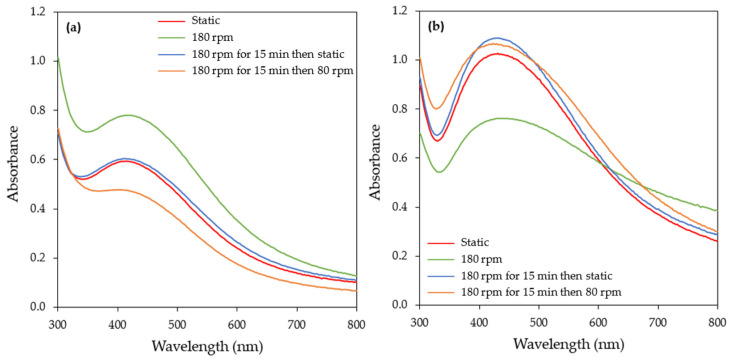
UV–visible absorption spectra of AgNPs biosynthesized by the CFS of *H. pluvialis* culture at different stirring conditions and for reaction times of (**a**) 1 h and (**b**) 24 h. (Conditions: illumination, T = 45 °C, pH = 11.0, AgNO_3_ concentration: 1 mM, AgNO_3_ aqueous solution to CFS ratio: 90:10 (*v*/*v*)).

Continuous stirring at 180 rpm increased the rate of AgNPs formation compared to the other stirring conditions tested (Figure 9 and Figure S6). According to collision theory, for the rate of a reaction, agitation allows metallic silver to attain the desired sites and increases the frequency of effective collisions, thus increasing the rate of reaction [57]. When increasing the time of the reaction (6 and 24 h), a decrease in the SPR band intensity was observed, followed by its broadening towards a longer wavelength (Figure 9b and Figure S6), which is an indication of the aggregation of the particles. Li and Kaner [58] reported that stirring induces the aggregation of nanoparticles. Under the other three stirring conditions, the reaction rate was almost similar, with a continuous increase in the SPR band intensity over time (Figure 9 and Figure S6). 

The results on the effect of stirring during the synthesis of AgNPs reported in the literature are contradictory. In the study of Chan and Don [59], the agitation speed was identified as one of the most influencing operating parameters during the synthesis of AgNPs by a cell-free filtrate of the white rot fungus *Pycnoporus sanguineus*. The authors reported that the size of the AgNPs decreased when the agitation speed of the reaction medium increased from 50 to 250 rpm. Contrary to the above, the agitation speed (50–200 rpm) had an insignificant effect on AgNPs production by the cell-free filtrate of *Trichoderma harzianum* SYA.F4 [60]. The synthesis of AgNPs by the cell-free extract of *Trichoderma reesei* was evaluated under static and dynamic conditions (agitation speed 50–250 rpm) by Gemishev et al. [57]. The formation of AgNPs was achieved under all conditions but larger particles were obtained under static conditions. Ebrahiminezhad et al. [53] reported that stirring or shaking prevented the synthesis of AgNPs by the CFS of a *C. vulgaris* culture.

#### 3.1.7. Summary of Optimal Conditions for the Synthesis of AgNPs by the CFS of *H. pluvialis* Culture

AgNPs biosynthesis by the CFS of the *H. pluvialis* culture exhibited a higher reaction rate under illumination conditions compared to darkness and thus all experiments were conducted under illumination. The effect of the temperature on the biosynthesis of AgNPs was investigated in a temperature range from 25 to 55 °C. Rapid AgNPs synthesis was observed at 55 °C, as indicated by the high value of the pseudo-first-order rate constant, but the UV–vis absorption spectra showed that as the reaction time increased, the aggregation of the nanoparticles occurred. Aiming at a high reaction rate as well as stable AgNPs, the temperature of 45 °C was selected as the optimum one. Five different pH levels (5, 7, 8, 9, and 11) were tested and pH = 11.0 proved to be the most effective in AgNPs synthesis. Various metal precursor (AgNO_3_) concentrations (1–5 mM) were evaluated and the results indicated that, for all time points tested, a linear relationship between the SPR band intensity and AgNO_3_ concentration was observed. Three AgNO_3_ aqueous solution tο CFS ratios (namely 95:5, 90:10, and 85:15 *v*/*v*) were studied, with 90:10 *v*/*v* being the most effective one. The synthesis of AgNPs was feasible under all stirring conditions tested. Continuous stirring at 180 rpm initially favored AgNPs synthesis but extending the reaction time resulted in the destabilization and aggregation of the nanoparticles, as indicated by the UV–vis absorption spectra. Concerning the other stirring conditions (static, stirring at 180 rpm for 15 min followed by static conditions, and stirring at 180 rpm for 15 min followed by stirring at 80 rpm), no significant differences were observed. 

The reaction time was found to be critical during the biosynthesis of AgNPs. A prolonged reaction time (up to 24 h) resulted in the broadening of the SPR band towards higher wavelengths, indicating the aggregation of the nanoparticles. Therefore, reaction times between 3 and 6 h are considered optimal for the synthesis of stable AgNPs.

The CFS of *H. pluvialis* offers an efficient alternative method for the synthesis of silver nanoparticles. The *H. pluvialis*-derived astaxanthin industry has been a commercial success for the nutraceutical market. Astaxanthin is accumulated inside *H. pluvialis* cells. Consequently, vast amounts of the culture supernatant are produced as a waste product, which results in environmental pollution [28]. The CFS contains various bioactive molecules that can act both as reducing and capping agents during AgNPs synthesis [32,33,35]. 

An extracellular polymeric substance (EPS) is complex mixture of macromolecules (proteins, carbohydrates, nucleic acids, and lipids) with varied functional groups, charges, and hydrophilicity. Being rich in reducing groups (such as hydroxyl, carboxyl, and uronic acids), microalgal polysaccharides can efficiently bind and reduce metal ions. Moreover, due to the dynamic supramolecular interactions facilitated by inter and intra-molecular hydrogen bonding, they can act as capping agents, providing stabilization and preventing further nanoparticle agglomeration [36]. Furthermore, according to Darwesh et al. [33], the extracellular release of nitrate-dependent reductase into the surrounding medium is considered to be one of the main factors for extracellular nanoparticle biosynthesis, especially for AgNPs. They are conjugated with an electron donor to reduce metal ions to the elementary form [33]. 

### 3.2. Characterization of Biosynthesized AgNPs

#### 3.2.1. Zeta Potential and Polydispersity Index (PDI) of the AgNPs

The zeta potential is an indicator of the surface charge of nanoparticles. Zeta potential (positive or negative) values indicate the tendency of the particles in a formulation to aggregate or to remain discrete. This is attributed to the electrostatic repulsion between particles with the same electric charge, which causes the segregation of the particles. According to the literature, nanoparticles with a zeta potential higher than +30 mV or lower than −30 mV are considered stable [61]. The polydispersity index (PDI) is a parameter that defines the heterogeneity of a sample based on its size. The PDI scale ranges from 0 to 1 (with 0 being monodisperse and 1 being polydisperse). It has been reported that samples with PDI values lower than 0.3 can be characterized as the monodisperse form [62]. 

The zeta potential as well as PDI values were measured in all cases after 24 h of reaction. The zeta potential values were found to be negative and in the range of −35.9 to −18.2 mV (Table 2), depending on the experimental conditions. The negative values of the zeta potential suggest that the capping agents on the surfaces of the AgNPs were electronegative. At pH values of 5.0 to 9.0, the average zeta potential was −27.6 ± 1.2 mV, while at pH = 11.0, the value increased to −18.2 ± 5.15 mV, indicating the moderate stability of the synthesized AgNPs. The average zeta potential of the AgNPs synthesized at a temperature range of 25 to 45 °C was −31.2 ± 1.4 mV, while, at the highest temperature (55 °C) examined, an increase in the zeta potential value (−20.6 ± 4.60 mV) was observed. Of the stirring conditions examined, all except continuous stirring at 180 rpm resulted in AgNPs with an average zeta potential of −34.1± 2.7 mV, denoting that the nanoparticles were stable (Table 2). In the case of a high temperature and continuous stirring at 180 rpm during the synthesis of AgNPs, for a reaction time greater than 3 h, the SPR band broadened towards a longer wavelength, indicating the aggregation of the particles. With an increasing metal precursor concentration (AgNO_3_) (higher than 3 mM) and AgNO_3_ aqueous solution tο CFS ratio, AgNPs with moderate stability were obtained. Under the optimal synthesis conditions, the zeta potential of the AgNPs was found to be −40.4 ± 8.41 mM, indicating highly stable AgNPs (Table 2, Figure S7). Negative values of the zeta potential were reported for AgNPs prepared by different microalgae. The zeta potential of the AgNPs synthesized by the cell-free extract of *C. vulgaris* was found to be −26.4 ± 4.22 mV [34], while the corresponding value for AgNPs synthesized by the EPS of *C. pyrenoidosa* was −12.16 ± 2.41 mV [36]. Furthermore, Hamida et al. [38] reported that AgNPs synthesized by the cell-free extract of *Coelastrella aeroterrestrica* were highly stable, with a zeta potential value of −33 mV. 

The PDI values ranged between 0.177 and 0.418 (Table 2). The estimated PDI values clearly suggest that the majority of AgNPs production occurred in the monodisperse phase, with minor amounts occurring in the polydisperse phase. The smallest PDI value (0.177) was observed at pH = 11, indicating the relatively narrow particle size distribution, while, under optimal synthesis conditions, the PDI value was 0.226.

#### 3.2.2. XRD Analysis

**Figure 10:**
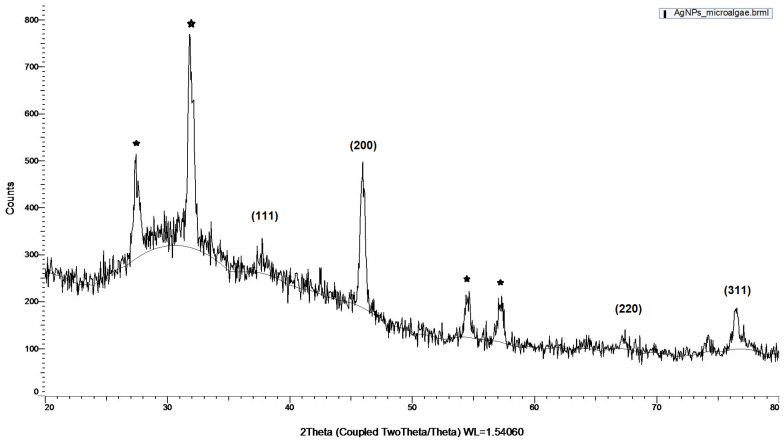
XRD patterns of AgNPs synthesized by the cell-free supernatant of *H. pluvialis* culture.

The crystalline nature of the synthesized AgNPs was confirmed by XRD under the 2θ full spectrum ranging from 20° to 80° (Figure 10). 

The Bragg reflection of the 2θ peaks was observed at 37.5°, 46.0°, 67.0°, and 76.5° and was, respectively, indexed to the planes of face-centered cubic (FCC) crystal lattice (111), (200), (220), and (311). The XRD outcome is consistent with the standards of the Joint Committee on Powder Diffraction (JCPDS No. 04-0783) [63]. The results of the present study are in accordance with others reported in the literature using *D. salina* [40]; the ethanolic extract of two microalgae belonging to the genus *Dictyosphaerium* [64]; the cell extract of *Spirulina platensis* [65]; the extract of the brown marine algae *C. moniliformis* [48]; and the cell-free extract of *C. vulgaris* [34]. In addition to the Bragg peaks representative of FCC silver nanocrystals, there are also some unassigned peaks marked with a star. These peaks may suggest that the crystallization of bio-organic phase occurs on the surface of the silver nanoparticles. In this study, the bio-organic phase consists of organic compounds secreted to the culture supernatant of the microalgae cells.

#### 3.2.3. TEM Analysis

**Figure 11:**
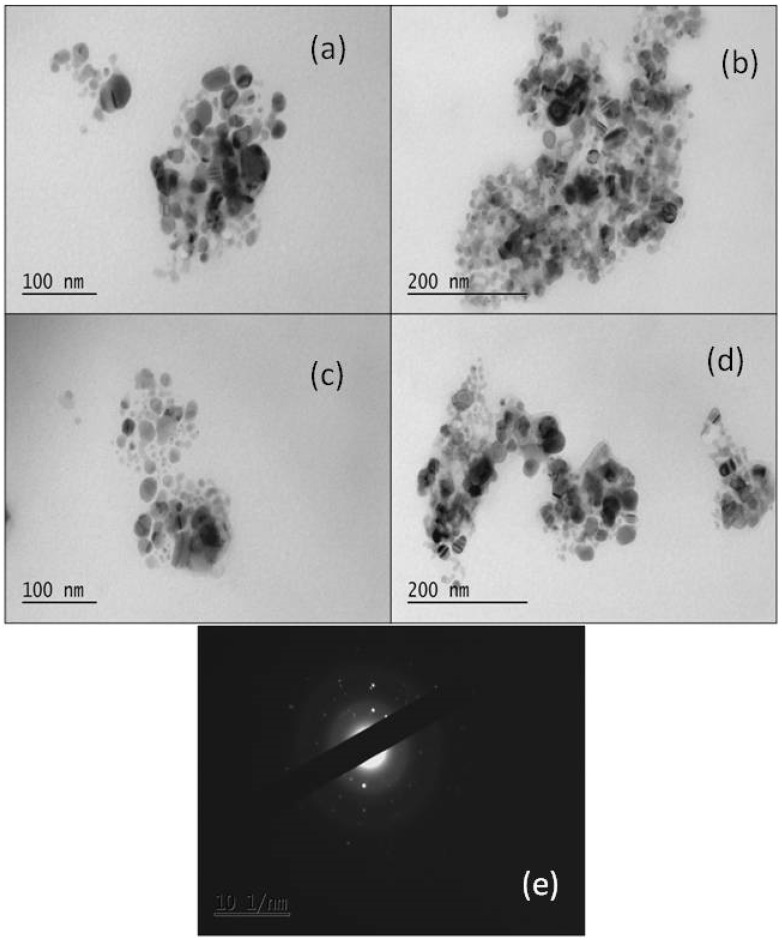
(**a**–**d**) TEM micrograph of the silver nanoparticles synthesized by the cell-free supernatant of *H. pluvialis* culture at different magnifications and (**e**) SAED pattern of the AgNPs.

Further insights into the morphology and the size of the synthesized nanoparticles were provided by TEM images (Figure 11). The size of the nanoparticles varied from 30 to 50 nm, with a quasi-spherical shape. 

The TEM images demonstrate the presence of nanoparticles inside organic structures, appearing as a shadowy layer around the particles. Nanoparticles are formed and/or trapped within organic structures, indicating that these components may act as reducing and stabilizing agents. The crystallinity of the synthesized nanoparticles, and not the amorphous structure, as previously confirmed by the XRD pattern, is also verified by the selected area electron diffraction pattern (SAED) (Figure 11e). 

The size and shape of the biosynthesized AgNPs strongly depend on the synthesis conditions as well as on the type of algae used (Table 1). In the study of Husain et al. [41], 30 freshwater cyanobacterial extracts were screened for their ability to synthesize AgNPs, under the same synthesis conditions. The size of the AgNPs was determined to be up to 50 nm, but the shape, either spherical, triangular, cubic, or pentagonal, was found to be dependent on the cyanobacterial strain (Table 1).

On the other hand, Moares et al. [37], using the aqueous extracts of two cyanobacteria (*Synechococcus elongatus* and *Microcystis aeruginosa*) and four green algae (*Coelastrum astroideum, Desmodesmus armatus, Cosmarium punctulatum,* and *Klebsormidium flaccidum*) as bioreducing agents, reported that the shape of the synthesized AgNPs was similar (spherical), but cyanobacterial extracts resulted in smaller-sized AgNPs compared to green algae ones (Table 1).

### 3.3. Long-Term Stability of the Biosynthesized AgNPs

The long-term stability of AgNPs depends on numerous factors, with the storage temperature, light exposure, and nature of the capping agent being the most important ones [66]. The production of AgNPs by applying green synthesis eliminates the need for a capping agent during synthesis, since the biological molecules can serve both as reducing and capping agents. The long-term stability of the AgNPs is critical since the loss of stability can lower their effectiveness in different applications. 

**Figure 12:**
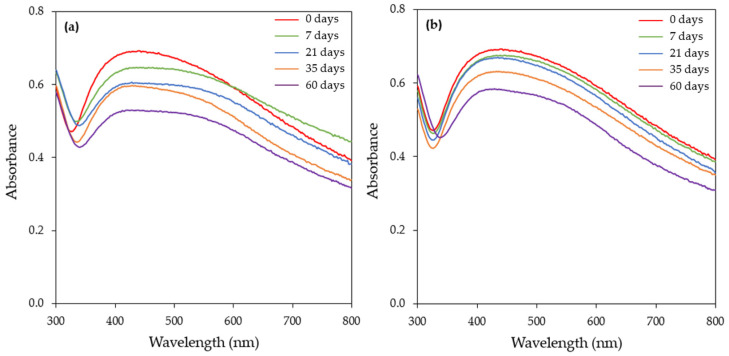
Effect of storage conditions on the stability of AgNPs. UV–visible absorption spectra of AgNPs stored in the dark at (**a**) ambient temperature and (**b**) 4 °C.

The stability of freshly prepared AgNPs was tested at ambient temperature and at 4 °C in the dark. The exposure of the AgNPs to light may induce fragmentation and subsequent fusion, resulting in morphology changes [47]. At different time intervals (0, 7, 21, 35, and 60 days), samples were withdrawn and the UV–vis spectrum and zeta potential were measured. The UV–vis spectra of the samples stored at ambient temperature showed a gradual reduction in the SPR band intensity over time. Furthermore, after one week of storage, the SPR band started to broaden towards a longer wavelength, which is an indication of the aggregation of the nanoparticles (Figure 12a). The zeta potential value on day 0 was −36.3 ± 7.77 mV, while, after 60 days of storage at ambient temperature, the value decreased to −30.8 ± 7.50 mV. Although the zeta potential was decreased (after 60 days of storage), based on the final value of the zeta potential, the AgNPs could be characterized as stable.

Figure 12b depicts the UV–vis spectra of the samples stored at 4 °C. As in the case of storage at ambient temperature, a decrease in the SPR band intensity was recorded over time. Contrary to ambient conditions, the SPR band started to broaden towards longer wavelengths after 60 days of storage (Figure 12b). At this time point, the zeta potential value was found to be −35.5 ± 7.49 mV. This value is slightly lower compared to the corresponding value on day 0 (−36.3 ± 7.77 mV), denoting better stability at 4 °C. The aforementioned experimental results show that the better long-term stability of the synthesized AgNPs can be achieved in darkness and at a low temperature.

Izak-Nau et al. [67] reported that the long-term stability of AgNPs mainly depends on their surface charge. They showed that the physicochemical properties of neutral particles did not change even when exposed to light at ambient temperature, while positively and negatively charged AgNPs agglomerated under the abovementioned experimental conditions, thus affecting their cytotoxicity. Velgosova et al. [68] also reported that negatively charged AgNPs, synthesized by the extract of the algae *Parachlorella kessleri*, were more stable when stored in darkness at 5 °C.

### 3.4. Antibacterial Activity of the Biosynthesized AgNPs

**Figure 13:**
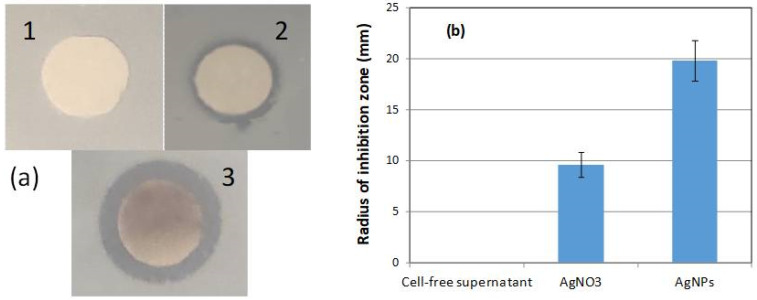
(**a**) Antibacterial activity test of AgNPs against *E. coli*. (1) Cell-free supernatant of *H. pluvialis culture*, (2) 1 mM AgNO_3_ aqueous solution, and (3) AgNPs. (**b**) Radius of inhibition zone for the samples (1), (2), and (3).

The antibacterial activity of the biosynthesized AgNPs against *E.coli* cells was investigated by the disc diffusion method. The CFS of the *H. pluvialis* culture did not exhibit any antibacterial activity (Figure 13a). On the other hand, the antimicrobial activity of silver has long been known. The radius of the inhibition zone in the case of the aqueous AgNO_3_ solution at a concentration of 1 mM was approximately 9.6 mm, while the inhibition potential of the AgNPs was much higher (19.8 mm) (Figure 13b). 

Tripathi and Goshisht [69] summarized the mechanisms linked to the antibacterial activity of AgNPs, i.e., (a) AgNPs adhere to the bacterial cell wall, disintegrate it, and, as a result, cell death occurs; (b) the disintegration of the cell membrane allows AgNPs to enter the cell, where they adversely affect DNA and proteins; (c) AgNPs can cause oxidative stress to the cells due to reactive oxygen species (ROS) and free radical generation; and (d) AgNPs putatively inflect cellular signaling. 

Similarly to the present study, AgNPs synthesized by *O. limnetica*, *C. aeroterrestrica*, *N. oleoabundans*, and *D. salina* exhibited antibacterial activity against *E. coli* [38,40,44,49].

## 4. Conclusions

In the present study, the extracellular synthesis of AgNPs was conducted using the cell-free supernatant (CFS) of a *H. pluvialis* culture under illumination conditions. The CFS supplied both reducing and stabilizing agents for the biosynthesis of nanoparticles. The effect of some synthesis conditions, such as the pH, temperature, concentration of the metal precursor, AgNO_3_ aqueous solution to CFS ratio, agitation conditions, and reaction time, was investigated. Alkaline conditions, a high temperature, and continuous agitation increased the rate of AgNPs production but prolonged the incubation time at the above conditions, which resulted in nanoparticles’ aggregation. The zeta potential measurements verified that AgNPs synthesized under the optimal conditions were highly stable. The long-term stability of the AgNPs was achieved in darkness and at a low temperature. The crystalline nature of the synthesized AgNPs was confirmed by XDR analysis, while the TEM analysis revealed that the AgNPs formed had a particle size of 30–50 nm and a quasi-spherical shape. Additionally, the biogenic AgNPs showed antibacterial activity against *E. coli*. The CFS of the *H. pluvialis* culture proved to be an efficient bioreducing agent for the production of stable AgNPs in an environmentally friendly and cost-effective manner.

## Figures and Tables

**Table 1:** Size and shape of AgNPs extracellularly synthesized under different conditions using various classes of algae.

Species	Synthesis Conditions	Shape	Size (nm)	Reference
*Chlamydomonas reinhardtii*	Bioreducing agent: EPS; C_AgNO3_ 1.25 mM; t_synthesis_ = 24 h; under illumination	Spherical	7.5	[39]
*Chlorella pyrenoidosa*	Bioreducing agent: EPS; C_AgNO3_ 3.5 mM; T = 90 °C; pH = 8.0; t_synthesis_ = 60 min	Spherical	5–15	[36]
*Chlorella vulgaris*	Bioreducing agent: cell-free extract; C_AgNO3_ 3.0 mM; T ambient; AgNO_3_ solution to cell-free extract ratio 8:2 (*v*/*v*); pH = 12.0; t_synthesis_ = 24 h	Spherical	55.06	[34]
*Coelastrella aeroterrestrica*	Bioreducing agent: aqueous cell extract; C_AgNO3_ 1.0 mM; T ambient; AgNO_3_ solution to aqueous cell extract ratio 90:10 (*v*/*v*); t_synthesis_ = 24 h; under illumination	Hexagonal	14.5	[38]
*Coelastrum astroideum, Desmodesmus armatus, Cosmarium punctulatum Klebsormidium flaccidum*/ *Synechococcus elongatus Microcystis aeruginosa*	Bioreducing agent: aqueous cell extract; C_AgNO3_ 1.0 mM; T ambient; AgNO_3_ solution to aqueous cell extract ratio 20:10 (*v*/*v*) t_synthesis_ = 20 h	Spherical	1.8–5.4/2.3–2.6	[37]
*Cylindrospermum stagnale NCCU*	Bioreducing agent: aqueous cell extract; C_AgNO3_ 1.0 mM; T = 30 °C; t_synthesis_ = 250 h; under illumination	Pentagonal	38–40	[41]
*Cystophora moniliformis* (brown marine algae)	Bioreducing agent: aqueous extract; C_AgNO3_ 1.0 mM; T = 65 °C; AgNO_3_ solution to aqueous cell extract ratio 90:10 (*v*/*v*); t_synthesis_ = 30 min	Spherical	50–100	[48]
*Dunaliella salina*	Bioreducing agent: aqueous cell extract; C_AgNO3_ 1.0 mM; T ambient; AgNO_3_ solution to aqueous cell extract ratio 50:1 (*v*/*v*); t_synthesis_ = 15 min	Spherical	35	[40]
*Dunaliella salina*	Bioreducing agent: aqueous cell extract; C_AgNO3_ 4.0 mM; pH = 7.0; T = 38 °C; AgNO_3_ solution to aqueous cell extract ratio 95:5 (*v*/*v*); t_synthesis_ = 35 min; under bright sunlight	Spherical	1–30	[42]
*Hapalosiphon fontinalis NCCU-339*	Bioreducing agent: aqueous cell extract; C_AgNO3_ 1.0 mM; T = 30 °C; t_synthesis_ = 270 h, under illumination	Triangular	50	[41]
*Microchaete* sp. *NCCU-342*	Bioreducing agent: aqueous cell extract; C_AgNO3_ 1.0 mM; T = 30 °C; t_synthesis_ = 30 h, under illumination	Spherical	40	[41]
*Neochloris oleoabundans*	Bioreducing agent: aqueous cell extract; C_AgNO3_ 0.8 mM; pH = 5.0; T = 27 °C; t_synthesis_ = 6 h, under illumination	Quasi-spherical	16.63	[43]
*Oscillatoria limnetica*	Bioreducing agent: aqueous cell extract; C_AgNO3_ 0.5 mM; pH = 6.7; T = 35 °C; AgNO_3_ solution to aqueous cell extract ratio 7:3 (*v*/*v*); t_synthesis_ = 48 h	Spherical	3.30–17.97	[49]
*Phormidium* sp. *NCCU-104*	Bioreducing agent: aqueous cell extract; C_AgNO3_ 1.0 mM; T = 30 °C; t_synthesis_ = 96 h; under illumination	Cubic	48	[41]
*Haematococcus pluvialis*	Bioreducing agent: CFS; C_AgNO3_ 1.0 mM; T = 55 °C; pH = 11.0; t_synthesis_ = 6 h; under illumination	Quasi-spherical	30–50	Present study

**Table 2:** Zeta potential and PDI of AgNPs synthesized under illumination by the cell-free supernatant of *H. pluvialis* culture at different experimental conditions after 24 h of reaction.

Experimental Conditions	PDI	Zeta Potential (mV)
**pH**		
5	0.335	−26.4 ± 5.9
7	0.291	−26.7 ± 8.1
8	0.361	−28.5 ± 6.7
9	0.258	−28.6 ± 6.3
11	0.177	−18.2 ± 5.2
**T (°C)**		
25	0.322	−30.5 ± 6.8
35	0.377	−30.3 ± 6.5
45	0.341	−32.9 ± 6.5
55	0.418	−20.6 ± 4.6
**C_AgNO3_ (mM)**		
1	0.377	−30.5 ± 5.95
2	0.377	−33.0 ± 7.95
3	0.31	−32.7 ± 5.22
4	0.329	−26.4 ± 5.85
5	0.356	−22.9 ± 5.00
**AgNO_3_ Aqueous Solution tο Cell-Free** **Supernatant Ratio (*v*/*v*)**		
95/5	0.313	−34.1 ± 9.31
90/10	0.322	−30.5 ± 6.84
85/15	0.332	−29.0 ± 5.50
**Stirring (rpm)**		
Stirring at 180 rpm for 15 min followed by stirring at 80 rpm	0.321	−31.0 ± 7.33
Stirring at 180 rpm for 15 min followed by static conditions	0.293	−35.4 ± 7.83
Static conditions	0.372	−35.9 ± 7.06
Continuous stirring at 180 rpm	0.270	−26.4 ± 4.70
Under optimal conditions	0.226	−40.4 ± 8.41

## Data Availability

The data are available upon reasonable request to the corresponding author.

## Supplementary Materials

The following supporting information can be downloaded at: https://www.mdpi.com/article/10.3390/ma17010187/s1, Figure S1: UV–visible absorption spectra of AgNPs biosynthesized by the CFS of *H. pluvialis* culture at different temperatures and for reaction time of (a) 0.25 h, (b) 3 h, (c) 6 h. (Conditions: illumination, pH = 8.0, AgNO_3_ concentration: 1 mM, AgNO_3_ aqueous solution to CFS ratio: 90:10 (*v*/*v*), stirring: 180 rpm for 15 min followed by stirring at 80 rpm); Figure S2. UV–visible absorption spectra of AgNPs biosynthesized by the CFS of *H. pluvialis* culture at various pHs and for reaction time of (a) 0.25 h, (b) 3 h, and (c) 6 h. (Conditions: illumination, T = 45 °C, AgNO_3_ concentration: 1 mM, AgNO_3_ aqueous solution to CFS ratio: 90:10 (*v*/*v*), stirring: 180 rpm for 15 min followed by stirring at 80 rpm; Figure S3. UV–visible absorption spectra of AgNPs biosynthesized by the CFS of *H. pluvialis* culture at various AgNO_3_ concentrations (1–5 mM) and for reaction time of (a) 1 h, (b) 3 h, (c) 6 h, and (d) 24 h. (Conditions: illumination, T = 45 °C, pH = 11.0, AgNO_3_ aqueous solution to CFS ratio: 90:10 (*v*/*v*), stirring: 180 rpm for 15 min followed by stirring at 80 rpm; Figure S4. Linear relationship between AgNO_3_ concentration and absorbance at λ_max_ (420 nm). Symbols: (●) 1 h reaction time $ABS\;(at\;\lambda_{max})=0.0987*C_{{AgNO}_{3}}+0.4065$, (R^2^ = 0.96, *p* = 0.0042), (○) 3 h reaction time $ABS\;(at\;\lambda_{max})=0.1162*C_{{AgNO}_{3}}+0.6392$, (R^2^ = 0.99, *p* = 0.0002), (▲) 6 h reaction time $ABS\;(at\;\lambda_{max})=0.1235*C_{{AgNO}_{3}}+0.7043$, (R^2^ = 0.97, *p* = 0.0026), (△) 24 h reaction time $ABS\;(at\;\lambda_{max})=0.0904*C_{{AgNO}_{3}}+0.8228$, (R^2^ = 0.97, *p* = 0.0018); Figure S5. UV–visible absorption spectra of AgNPs biosynthesized by the CFS of *H. pluvialis* culture at different AgNO_3_ aqueous solution to CFS ratios and for reaction time of (a) 0.25 h, (b) 1 h, and (c) 6 h; Figure S6. UV–visible absorption spectra of AgNPs biosynthesized by the CFS of *H. pluvialis* culture at various stirring conditions and for reaction time of (a) 0.25 h, (b) 3 h, and (c) 6 h. (Conditions: illumination, T = 45 °C, pH = 11.0, AgNO_3_ concentration: 1 mM, AgNO_3_ aqueous solution to CFS ratio: 90:10 (*v*/*v*)); Figure S7. Zeta potential of AgNPs biosynthesized by the CFS of *H. pluvialis* culture under optimal conditions.
