# Supplementary material for: Green Synthesis of Silver Nanoparticles Using the Cell-Free Supernatant of Haematococcus pluvialis Culture
Source: Materials (Basel). 2023 Dec 29;17(1):187. doi: 10.3390/ma17010187 (PMC10779655; doi:10.3390/ma17010187)
Supplement: Supplementary file 1 [file materials-17-00187-s001.zip › materials-2715054-supplementary.pdf]

Green synthesis of silver nanoparticles using the cell-free supernatant of *Haematococcus pluvialis* culture

Maria G. Savvidou[1,2†], Evgenia Kontari[1†], Styliani Kalantzi[1], Diomi Mamma[1*]

[1] Biotechnology Laboratory, School of Chemical Engineering, National Technical University of Athens, 9 Iroon Polytechniou Str, Zografou Campus, 15700, Athens Greece

[2] Current affiliation: Department of Biomedical Engineering, Tufts University, Medford, 02155, MA, USA

*Corresponding author, Tel: +30 210 7723160, Fax: +30 210 7723161,
e-mail address: dmamma@chemeng.ntua.gr

† These authors contributed equally to this paper

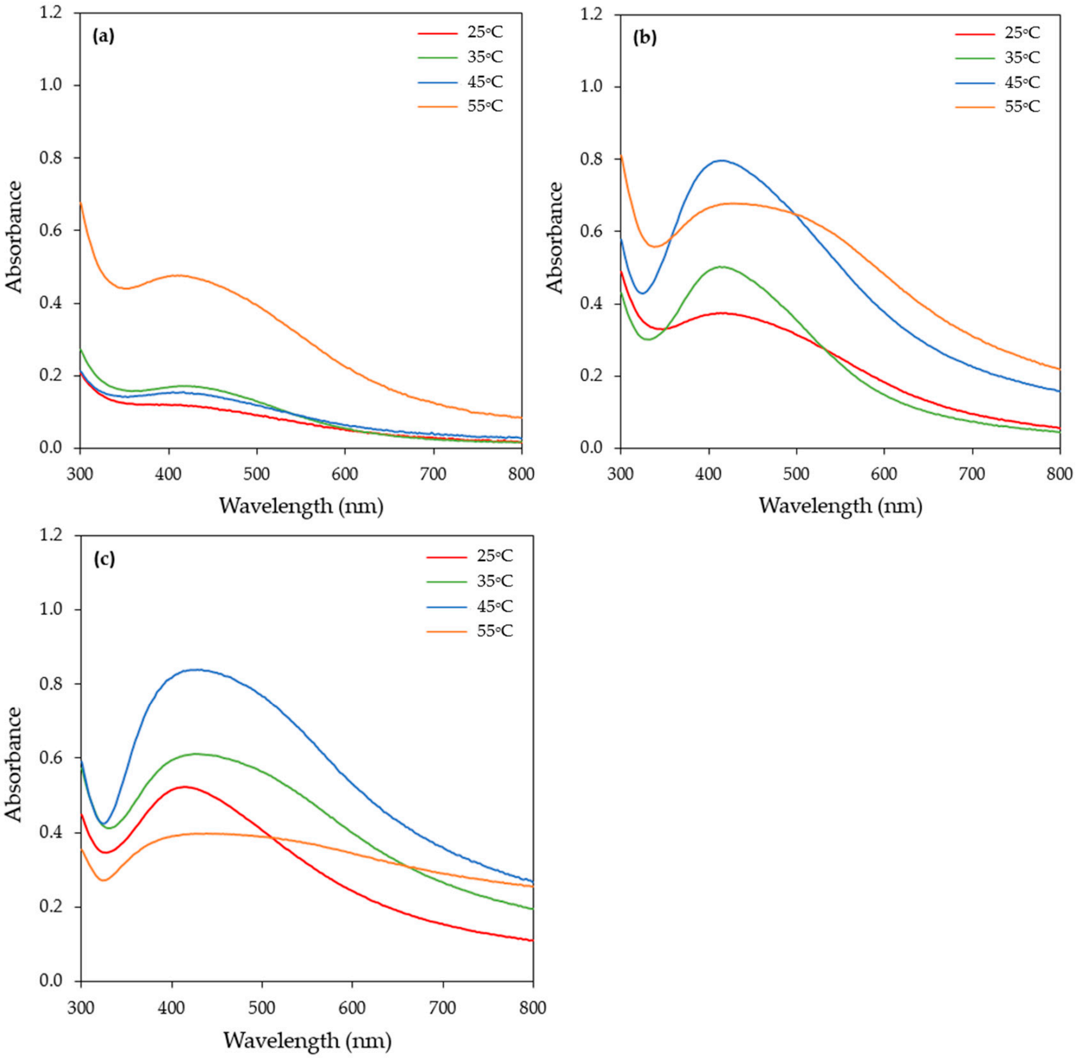

Supplementary Figure S1. UV–visible absorption spectra of AgNPs biosynthesized by the CFS of *H. pluvialis* culture at different temperatures and for reaction time of (a) 0.25 h, (b) 3 h, (c) 6h. (Conditions: illumination, pH=8.0, $AgNO_3$ concentration: 1mM, $AgNO_3$ aqueous solution to CFS ratio: 90:10 (v/v), stirring: 180 rpm for 15 min followed by stirring at 80 rpm)

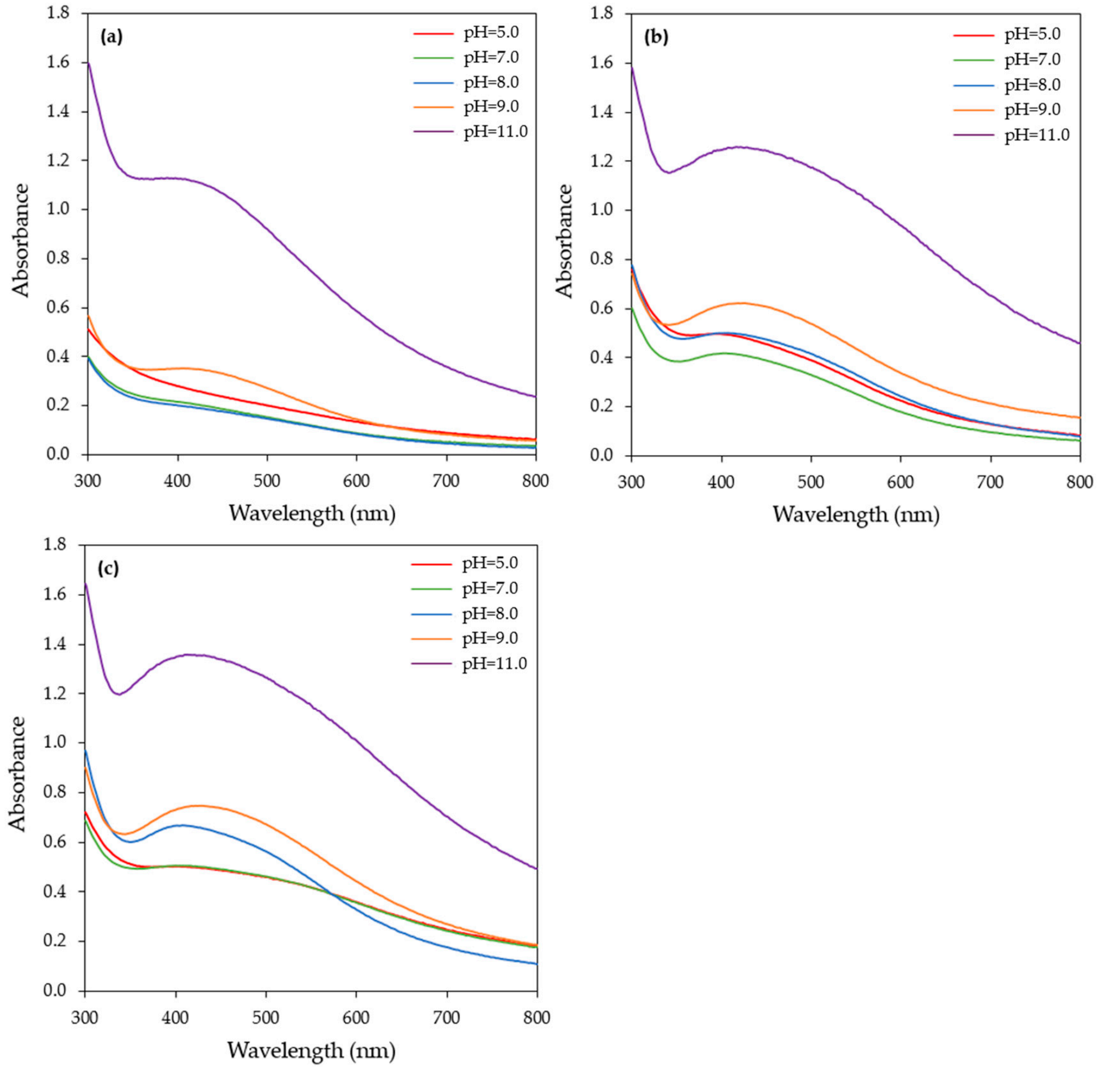

Supplementary Figure S2. UV–visible absorption spectra of AgNPs biosynthesized by the CFS of *H. pluvialis* culture at various pHs and for reaction time of (a) 0.25 h, (b) 3 h, and (c) 6 h. (Conditions: illumination, T=45°C, $AgNO_3$ concentration: 1mM, $AgNO_3$ aqueous solution to CFS ratio: 90:10 (v/v), stirring: 180 rpm for 15 min followed by stirring at 80 rpm

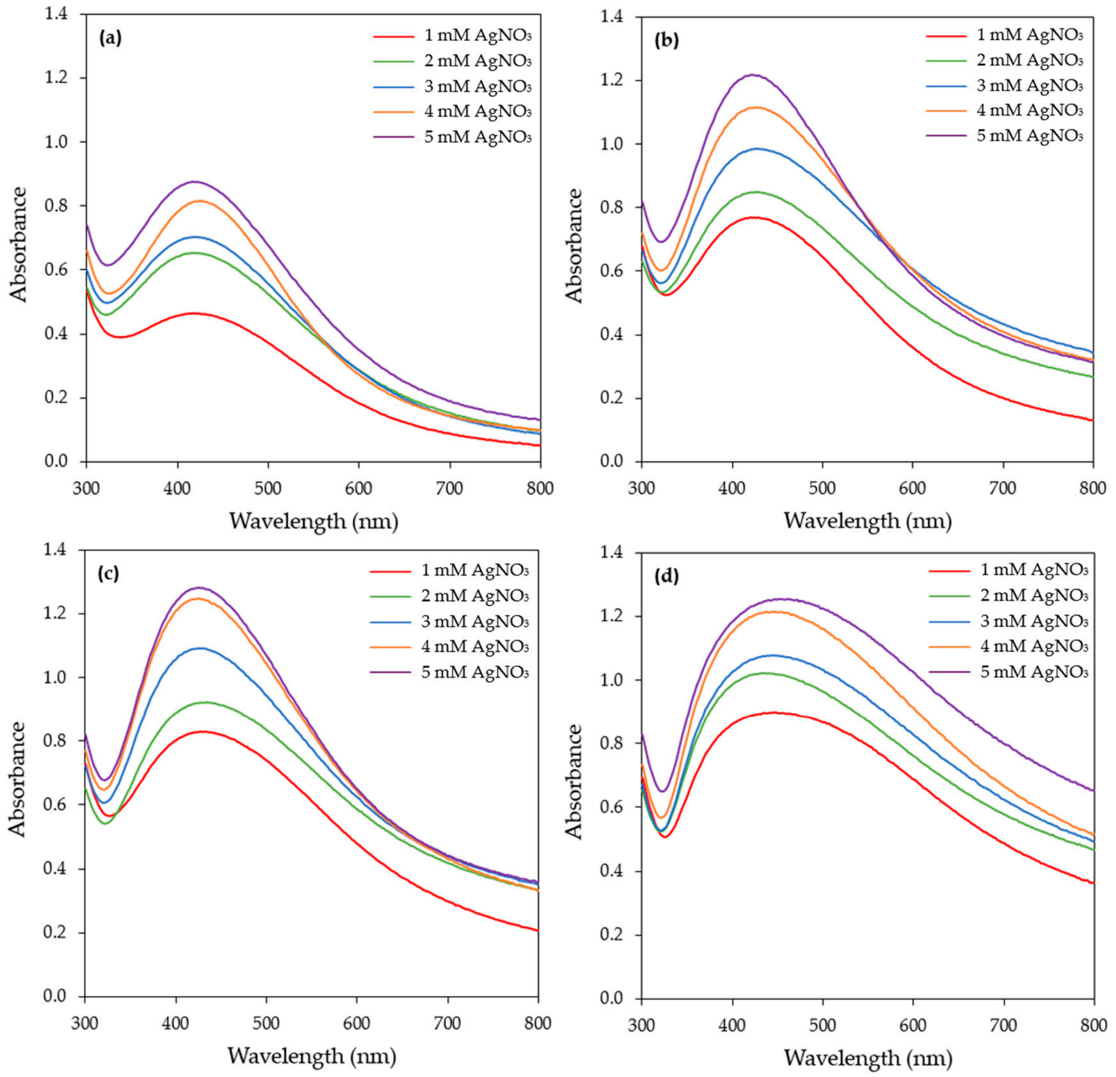

Supplementary Figure S3. UV–visible absorption spectra of AgNPs biosynthesized by the CFS of *H. pluvialis* culture at various $AgNO_3$ concentrations (1-5 mM) and for reaction time of (a) 1 h, (b) 3 h, (c) 6 h, and (d) 24 h. (Conditions: illumination, T=45°C, pH=11.0, $AgNO_3$ aqueous solution to CFS ratio: 90:10 (v/v), stirring: 180 rpm for 15 min followed by stirring at 80 rpm

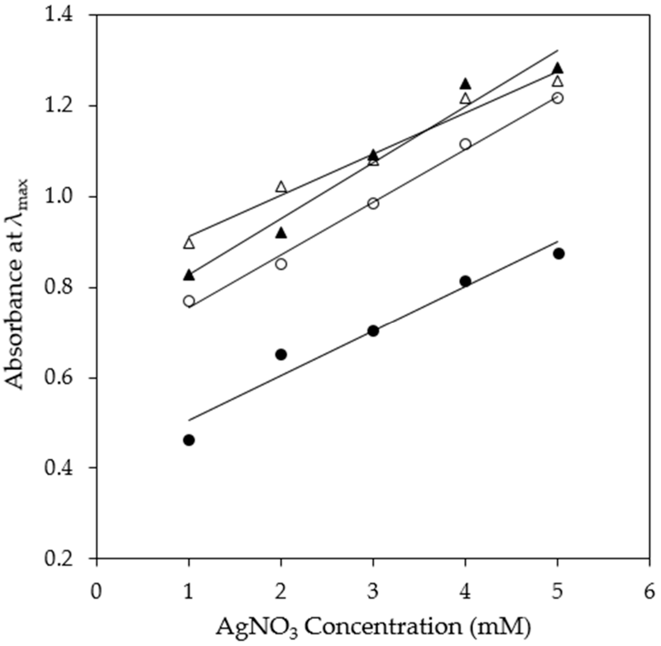

Supplementary Figure S4. Linear relationship between $AgNO_3$ concentration and absorbance at λ_{max} (420 nm). Symbols:

(●) 1h reaction time $ABS\ (at\ \lambda_{max}) = 0{,}0987 * C_{AgNO_3} + 0{,}4065$, ($R^2$=0.96, p=0.0042);

(○) 3 h reaction time $ABS\ (at\ \lambda_{max}) = 0{,}1162 * C_{AgNO_3} + 0{,}6392$, ($R^2$=0.99, p=0.0002);

(▲) 6 h reaction time $ABS\ (at\ \lambda_{max}) = 0{,}1235 * C_{AgNO_3} + 0{,}7043$, ($R^2$=0.97, p=0.0026);

(△) 24 h reaction time $ABS\ (at\ \lambda_{max}) = 0{,}0904 * C_{AgNO_3} + 0{,}8228$, ($R^2$=0.97, p=0.0018).

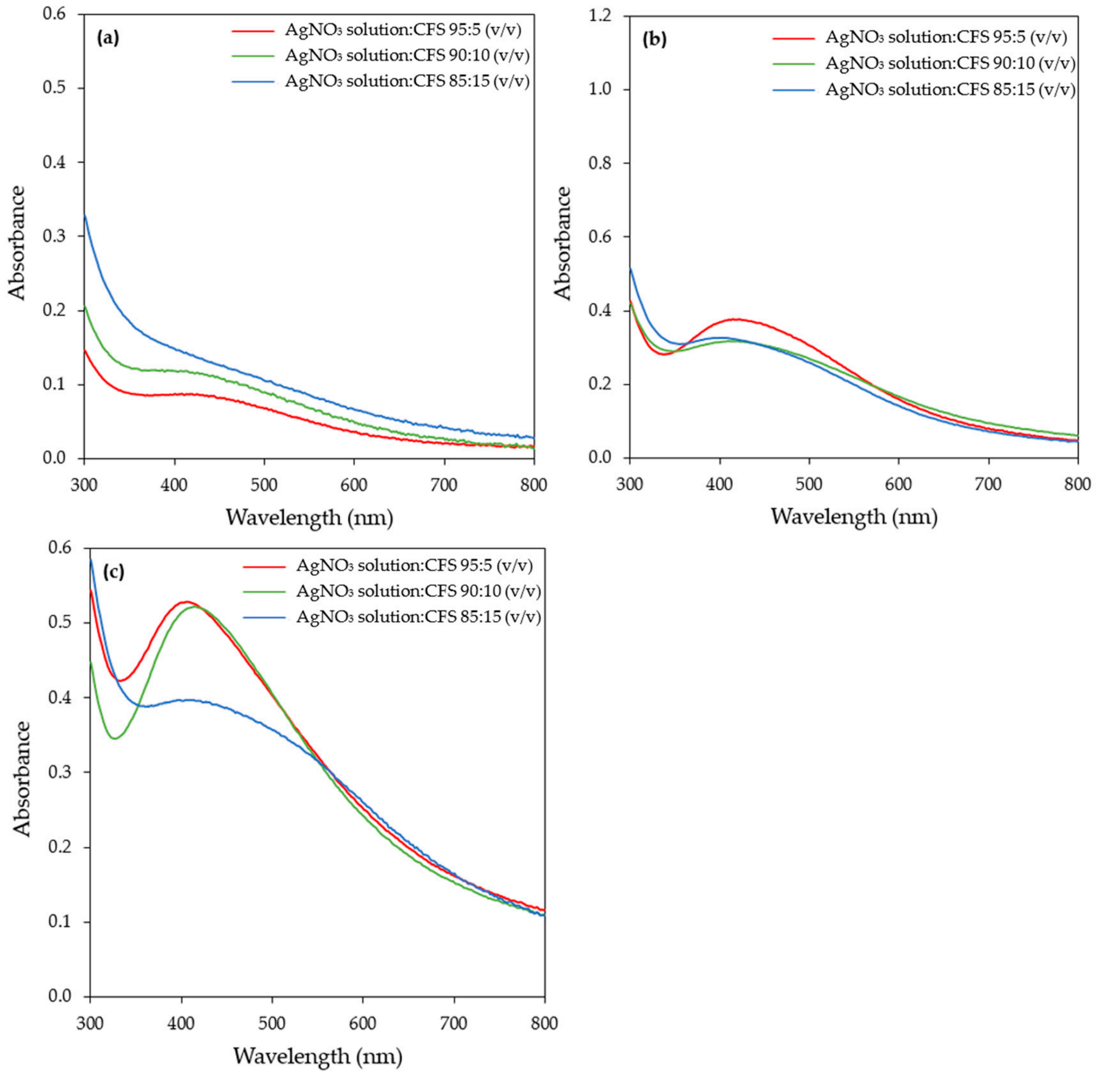

Supplementary Figure S5. UV–visible absorption spectra of AgNPs biosynthesized by the CFS of *H. pluvialis* culture at different $AgNO_3$ aqueous solution to CFS ratios and for reaction time of (a) 0.25 h, (b) 3 h, and (c) 6 h.

(Conditions: illumination, T=45°C, pH=11.0, $AgNO_3$ concentration: 1mM, stirring: 180 rpm for 15 min followed by stirring at 80 rpm).

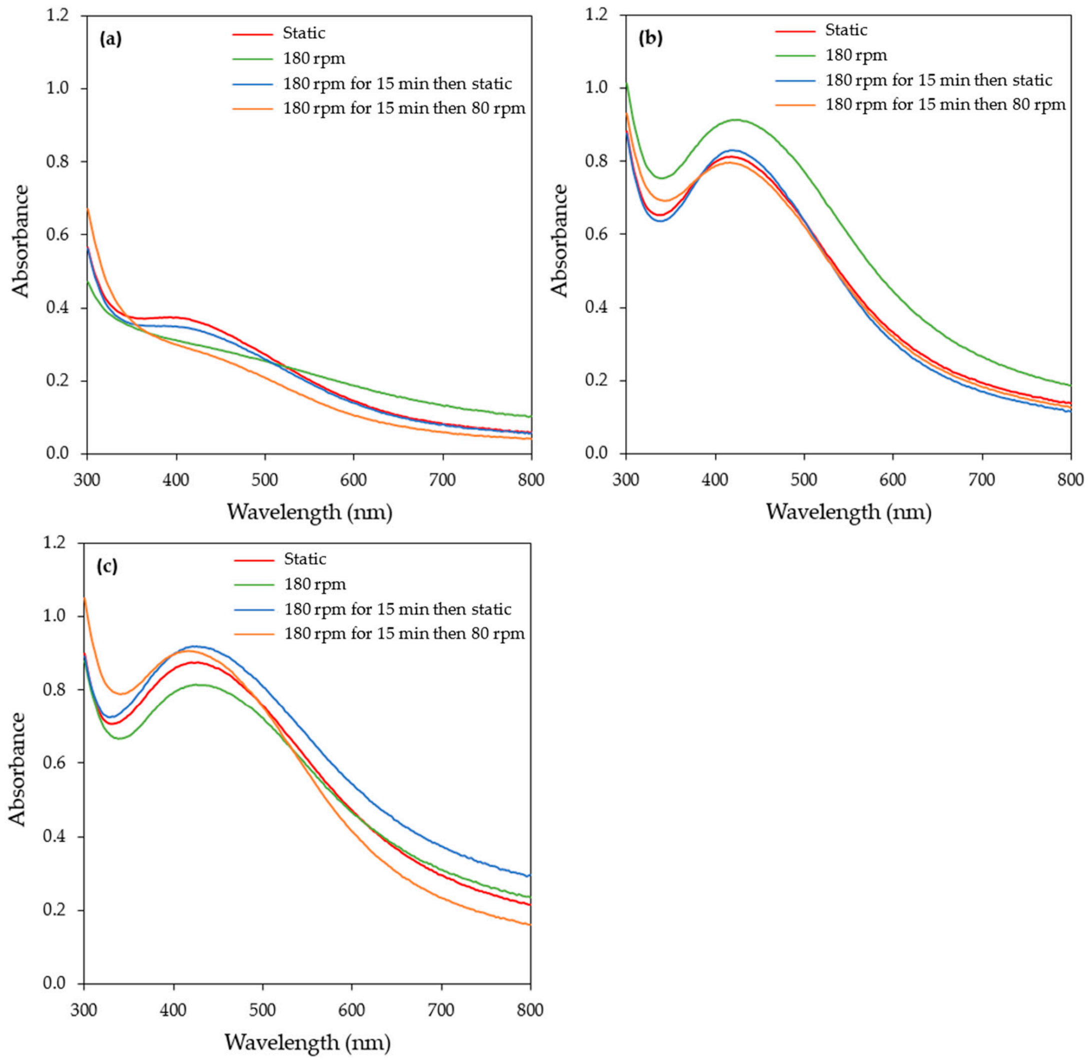

Supplementary Figure S6. UV–visible absorption spectra of AgNPs biosynthesized by the CFS of *H. pluvialis* culture at various stirring conditions and for reaction time of (a) 0.25 h, (b) 3 h, and (c) 6 h. (Conditions: illumination, T=45°C, pH=11.0, $AgNO_3$ concentration: 1mM, $AgNO_3$ aqueous solution to CFS ratio: 90:10 (v/v)).

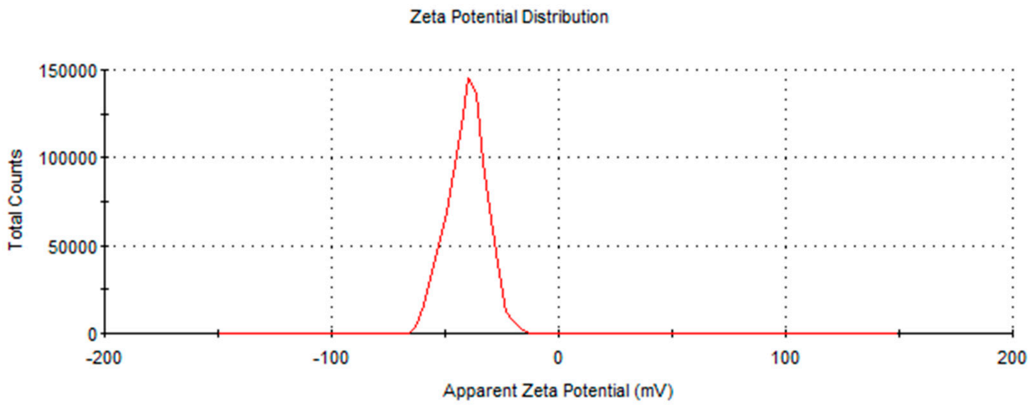

Supplementary Figure S7. Zeta potential of AgNPs biosynthesized by the CFS of *H. pluvialis* culture under optimal conditions.
